# Feasibility of single-isocenter, multi-arc non-coplanar volumetric modulated arc therapy for multiple brain tumors using a linear accelerator with a 160-leaf multileaf collimator: a phantom study

**DOI:** 10.1093/jrr/rru042

**Published:** 2014-06-18

**Authors:** Yoshio Iwai, Shuichi Ozawa, Tatsuya Ageishi, Roberto Pellegrini, Kiyoshi Yoda

**Affiliations:** 1 Research Physics, Elekta KK, 3-9-1 Shibaura, Minato-ku, Tokyo 108-0023, Japan; 2 Department of Radiation Oncology, Institute of Biomedical and Health Science, Hiroshima University, 1-2-3 Kasumi, Minami-ku, Hiroshima 734-8551, Japan; 3 Toshiba Medical Systems Corporation, 138 Shimoishigami, Otawara-shi, Tochigi 324-8550, Japan; 4 Elekta, via Bernardo Rucellai 23, Milano IT-20126, Italy

**Keywords:** radiosurgery, brain metastasis, single isocenter, VMAT, non-coplanar, film dosimetry, Agility, Monaco

## Abstract

The feasibility of single isocenter, multi-arc non-coplanar volumetric modulated arc therapy (VMAT) for multiple brain tumors was studied using an Elekta Synergy linear accelerator with an Agility multileaf collimator and a Monaco treatment planning system. Two VMAT radiosurgery plans consisting of a full arc and three half arcs were created with a prescribed dose of 20 Gy in a single fraction. After dose delivery to a phantom, ionization chambers and radiochromic films were used for dose measurement. The first VMAT radiosurgery plan had nine targets inside the phantom, and the doses were measured by the chambers at two different points and by the films on three sagittal and three coronal planes. The differences between the calculated dose and the dose measured by a Farmer ionization chamber and a pinpoint ionization chamber were <1.00% and <2.30%, respectively, and the average pass rates of gamma indices among the six planes under each of 3%/3 mm and 2%/2 mm criteria were 98.6% and 92.6%, respectively. The second VMAT radiosurgery plan was based on a clinical 14 brain metastases. Differences between calculated and film-measured doses were evaluated on two sagittal planes. The average pass rates of the gamma indices on the planes under each of 3%/3 mm and 2%/2 mm criteria were 97.8% and 88.8%, respectively. It was confirmed that single-isocenter, non-coplanar multi-arc VMAT radiosurgery for multiple brain metastases was feasible using Elekta Synergy with Agility and Monaco treatment planning systems. It was further shown that film dosimetry was accurately performed for a dose of up to nearly 25 Gy.

## INTRODUCTION

Linear-accelerator based stereotactic radiosurgery (SRS) has been widely employed for treating multiple brain tumors using conformal arc therapy (CAT) with multiple isocenters, where the treatment delivery time is roughly proportional to the number of tumors [1]. Prolonged treatment time may lead to tumor movement and thus reduced tumor control [2]. To decrease the SRS treatment time for multiple metastases, multi-isocenter volumetric modulated arc therapy (VMAT) has been used instead of multi-isocenter CAT [3, 4]. Wolff *et al*. compared SRS treatment deliveries between multi-isocenter single-arc VMAT and 5-arc CAT, demonstrating that VMAT provided much quicker delivery with increased mean dose to healthy brain [3]. Mayo *et al*. created non-coplanar multi-arc (2–3 arcs per isocenter) VMAT plans and treated 12 patients after validating dose distributions by using a 2D-array detector and films in a water-equivalent phantom [4].

In order to further shorten the treatment time of SRS for multiple metastases, single isocenter VMAT was evaluated [5–8]. Lagerwaard *et al*. compared single arc VMAT SRS with multiple isocenter multi-arc dynamic CAT, reporting that the treatment time was highly decreased and film measurements agreed with calculations [5]. They also studied single isocenter, coplanar 2-arc VMAT plans for three patients with multiple brain metastases, by way of simultaneous integrated boost with whole brain radiotherapy [6]. Meanwhile, Clark *et al*. reported that coplanar VMAT with a single isocenter had a significant planning limitation and that multi-arc non-coplanar VMAT with a single isocenter resulted in more favorable dose–volume histograms for challenging cases [7, 8]. However, the author focused on planning studies and did not show any dose verification results.

Recently, a new 160-leaf multileaf collimator (MLC) with a leaf width of 5 mm was developed by Elekta [9], but dosimetric aspects of VMAT SRS for multiple brain tumors using this MLC have not been published. The purpose of this paper was to study the feasibility of single isocenter, multi-arc non-coplanar VMAT SRS for multiple brain tumors using an Elekta Synergy linear accelerator with the 160-leaf MLC in terms of planning and dose verification.

## MATERIALS AND METHODS

Single-isocenter non-coplanar VMAT radiosurgery plans were created with a full arc (couch angle: 0°) and three half arcs (couch angles: ± 45° and 90°) using Monaco ver. 3.30 (Elekta AB, Stockholm, Sweden) based on X-ray Voxel Monte Carlo (XVMC) and constraint optimization algorithms with biological cost functions. A photon energy of 6 MV was employed throughout this study. The dose calculation grid size and the calculated dose statistics were set to 2 mm and 0.5% (normalized standard deviation) per plan, respectively.

**Fig. 1.**
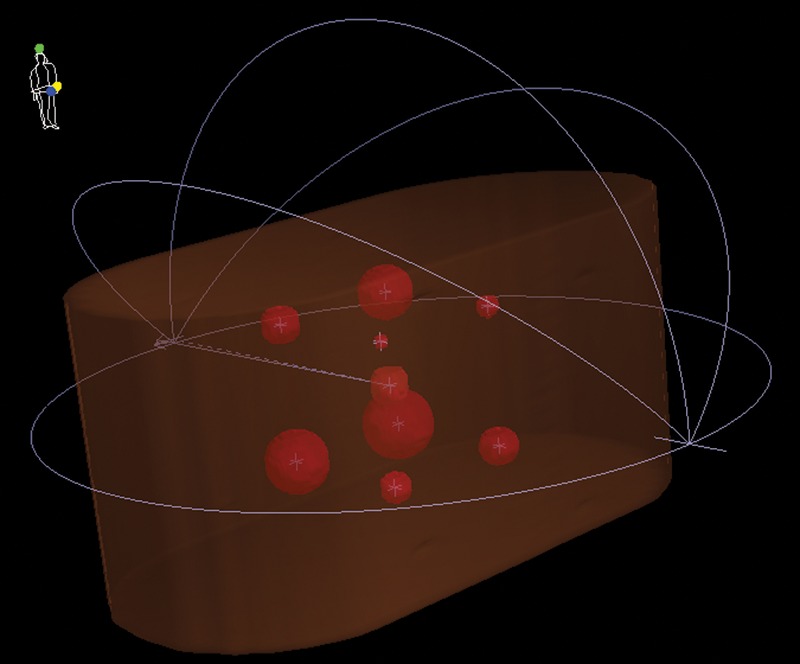
3D phantom view for a single-isocenter nine-target VMAT radiosurgery plan with a full arc and three half arcs. The planned nine targets were spheres with diameters ranging from 5–40 mm and located at the center and eight corners of a virtual cube having a side length of 8 cm, where the cube center and the center of an I'mRT phantom coincided. The linear accelerator isocenter was also positioned at the center of the phantom.

The first VMAT radiosurgery plan employed a nine spherical target model created inside an I'mRT phantom (IBA Dosimetry, Schwarzenbruck, Germany). The nine spherical targets had diameters ranging from 5–40 mm located at the center and at the eight corners of a virtual cube with an 80 mm side length. The center of the cube and the center of the phantom were coincident. Figure 1 indicates the positions of the nine targets as well as the beam arrangement of the plan. A dose of 20 Gy in a single fraction was prescribed for 95% of the planning target volume (PTV), and the isocenter was positioned at the center of the phantom. The Elekta Synergy linear accelerator with an Agility MLC (Elekta AB, Stockholm, Sweden) installed at the Radiation Therapy Training Center (RTTC) of Toshiba Medical Systems (Nasu, Tochigi, Japan) was used. The dose delivery for the nine-target plan was verified using ionization chambers and Gafchromic EBT3 films (Ashland Inc., Covington, USA). Point doses were measured at the isocenter (0 mm, 0 mm, 0 mm) and at the center of the largest target (40 mm, −40 mm, −40 mm) using 0.015 cm^3^ pinpoint and 0.6 cm^3^ Farmer ionization chambers (Type N31014 and Type N30013, PTW, Freiburg, Germany). Absolute dose distributions were measured using films on three parallel planes (either sagittal or coronal), including isocentric planes with a film interval of 40 mm. Prior to the film measurement, a dose ranging from 0–25 Gy was delivered to the film for calibration, where the films were oriented perpendicular to the beam axis and placed between two solid water phantoms, each with a thickness of 10 cm. The source-to-film distance was 100 cm.

**Fig. 2.**
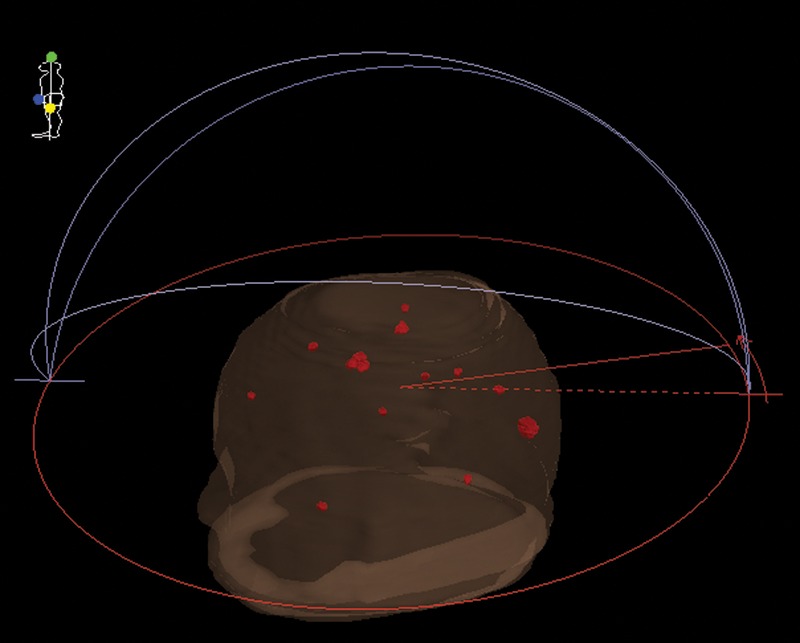
3D head view for a single-isocenter clinical 14 brain metastases (14-mets) VMAT radiosurgery plan with a full arc and three half arcs. The target volumes ranged from 0.03–0.71 cm^3^.

The second VMAT radiosurgery plan was based on clinical 14 brain metastases (14-mets) having the same arc beam geometry as the nine-target plan, except for the plan isocenter being positioned at the gravitational center of the 14 targets. The target volume was 0.12 cm^3^ on average, and ranged from 0.03–0.71 cm^3^. Figure 2 shows the target positions and the beam arrangement of this plan. A dose (D_95_) of 20 Gy in a single fraction was prescribed for 95% of each of the 14 PTVs with quadratic overdose constraints. For comparison, the same D_95_ dose was also prescribed for the entire 14 PTVs. The doses outside each PTV were constrained to 9, 6 and 2.5 Gy at a distance of 3, 6 and 20 mm from each PTV surface, respectively. Paddick's conformity index (PCI) and gradient index (PGI) were calculated for the 14-mets plan as a function of the target volume [10, 11].

To verify the 14-mets plan, film dosimetry was performed again by using the I'mRT phantom and the Gafchromic EBT3 films. With the isocenter and the center of the phantom coincident, films were positioned on two sagittal planes at −20 mm and −45 mm from the isocenter, where the dosimetry for six and four targets were evaluated, respectively.

The films were scanned in landscape orientation [using an ES-8500 (EPSON, Nagano, Japan) scanner with a spatial resolution of 75 dpi] after 15 h of irradiation with warm-up scans repeated three times. The scanned images were saved in 48-bit RGB tiff format. The green channel of the images was analyzed using SNC Patient software (Sun Nuclear, Melbourne, Florida, USA). For absolute dose distributions, gamma analyses under 3%/3 mm and 2%/2 mm criteria, with global percent differences were used with a dose threshold of 30% of the maximum measured dose on each film plane.

## RESULTS

**Fig. 3.**
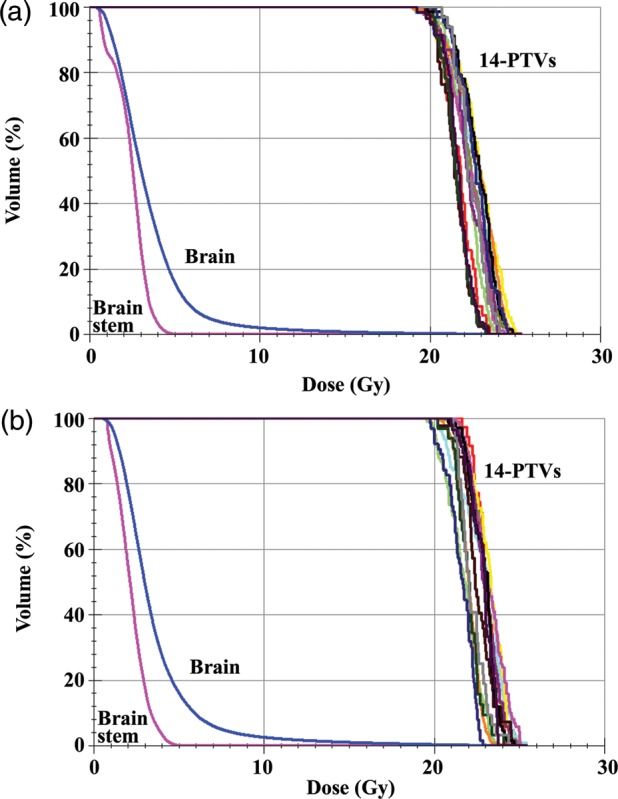
Dose–volume histograms. A D_95_ dose of 20 Gy in a single fraction was prescribed for (**a**) each of the 14 PTVs and (**b**) the entire volumes of the 14 PTVs. Dose restrictions were applied to normal brain, including the brain stem outside the targets.

Figure 3a and b show dose–volume histograms for the 14-mets plan, where a D_95_ dose of 20 Gy in a single fraction was prescribed for (Fig. 3a) each of the 14 PTVs and (Fig. 3b) the entire volumes of the 14 PTVs. As anticipated, the variation of D_95_ doses among the 14 PTVs was smaller when the dose was prescribed for each PTV. Further investigation would be required to reduce this variation.

Table 1 indicates the point doses measured by the ionization chambers along with the percentage dose differences between the Monaco calculations and the chamber measurements for the nine-target plan. The dose differences between the calculations and the measurements using the Farmer and the pinpoint ionization chambers were <1.00% and <2.30%, respectively.

**Table 1.** A comparison of measured and calculated doses for the nine-target plan using Farmer (N30013) and pinpoint (N31014) chambers

Position (mm)	Target diameter (mm)	Chamber	Measurement (Gy)	Calculation (Gy)	Difference
(0, 0, 0)	20	Farmer pinpoint	22.89 24.73	22.68 24.19	0.93% 2.23%
(40, −40, −40)	40	Farmer pinpoint	21.59 21.11	21.75 21.54	−0.74% −2.00%

**Fig. 4.**
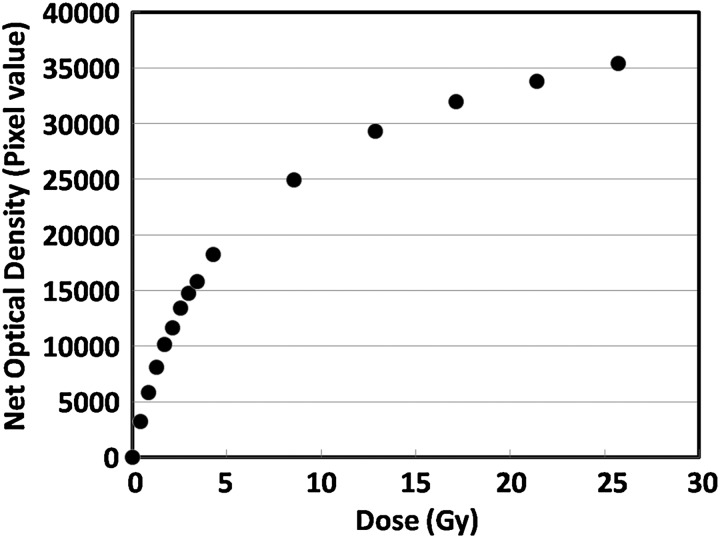
Plot of net optical density measured by a scanner green channel as a function of delivered dose. The films were oriented perpendicular to the beam axis and placed between two solid water phantoms each having a thickness of 10 cm. The source-to-film distance was 100 cm.

Figure 4 shows a plot of net optical density measured using the scanner green channel (16-bit depth) as a function of delivered doses, which agrees with the results reported by Casanova Borca *et al*. [12]. Table 2 indicates the pass rates of the absolute dose gamma analyses for the nine-target plan using the EBT3 films on six different planes. Average pass rates for the six planes under each of the 3%/3 mm and 2%/2 mm criteria were 98.6% (range, 99.4–97.6%) and 92.6% (range, 95.3–86.5%), respectively.

**Table 2.** The pass rates of the absolute dose gamma analyses for the nine-target plan under 3%/3 mm and 2%/2 mm (in brackets) criteria using EBT3 films, where a dose of 20 Gy in a single fraction was prescribed for 95% of the planning target volume

Sagittal film position (mm)	3%/3 mm (2%/2 mm)	Coronal film position (mm)	3%/3 mm (2%/2 mm)
+40	98.8% (95.3%)	+40	99.4% (94.6%)
0	99.2% (94.1%)	0	98.2% (86.5%)
−40	97.6% (90.5%)	−40	98.6% (94.3%)

A global percent difference criterion was employed with a dose threshold of 30% of the maximum measured dose on each film plane, where each film position was indicated as a distance from the isocenter plane.

**Fig. 5.**
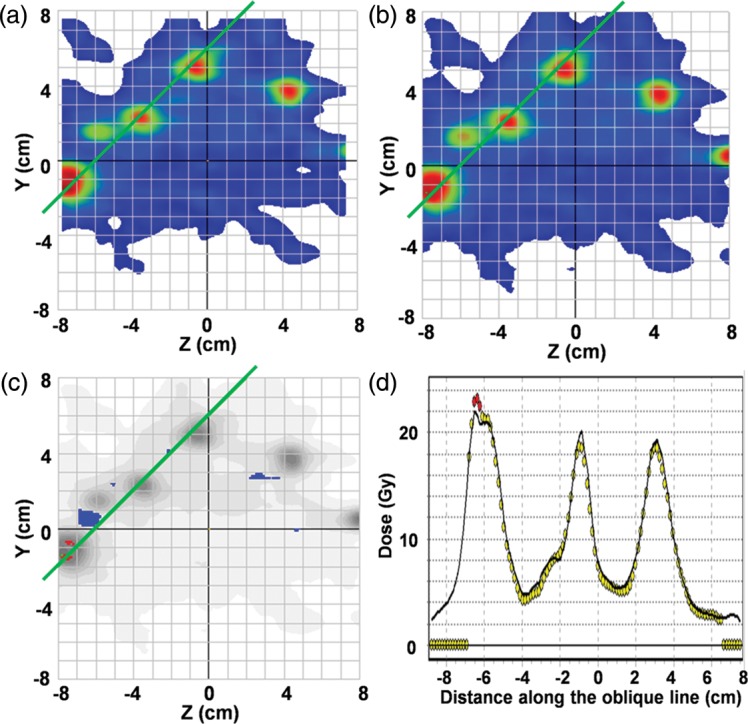
Film analysis result on a sagittal plane at −20 mm for the 14-mets plan: (**a**) a colored map of a measured dose distribution, (**b**) a colored map of a calculated dose distribution, (**c**) an absolute dose gamma plot under a 3%/3 mm criterion with a dose threshold of 30% of the maximum measured dose on the film plane, and (**d**) a comparison of the dose on an oblique green line indicated in Figs 5a–c. Circles and line show measurement and calculation, respectively.

For the 14-mets plan, average pass rates on the two sagittal planes (−20 mm and −45 mm from the isocenter) under each of the 3%/3 mm and 2%/2 mm criteria were 97.8% (range, 99.1–96.5%) and 88.8% (range, 90.1–87.4%), respectively. Figure 5 indicates the film dosimetry results on a sagittal plane at −20 mm for the 14-mets plan. The absolute film dosimetry agrees well with the Monaco calculation with a visible but small discrepancy detected at the left peak in Fig. 5d, the cause being unknown. The delivery time of the 14-mets plan was 40 min including couch rotation.

## DISCUSSION

**Fig. 6.**
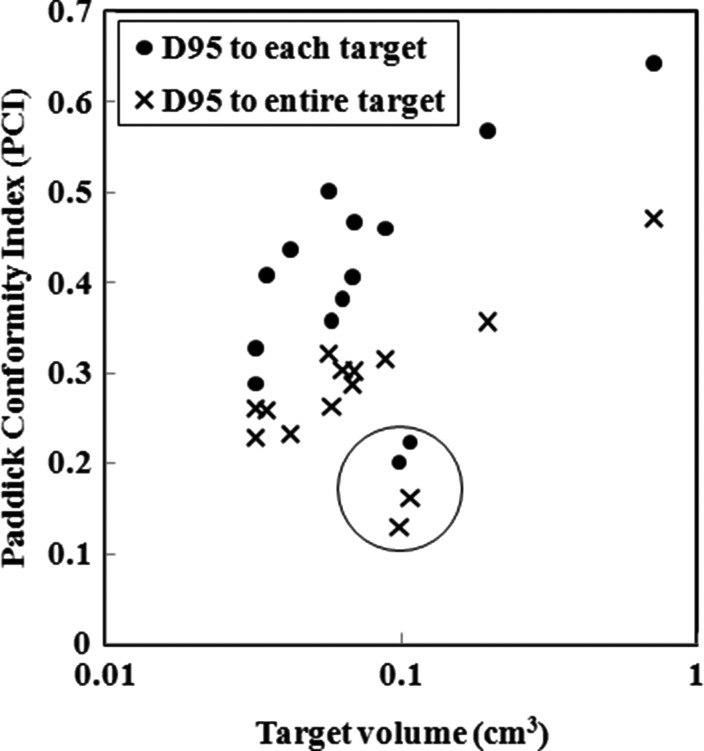
Plots of Paddick's conformity index (PCI) for the 14-mets plan as a function of the target volume, where D_95_ was prescribed for each of the PTVs (marked with filled circles) and to the entire PTVs (marked with X's). It was found that the PCIs for two targets were significantly lower than for the others (marked with open circle), possibly resulting from an exceptionally short distance between the two targets (1 cm).

Figure 6 shows plots of the PCI for the 14-mets plan as a function of the target volume, where D_95_ was prescribed for each of the PTVs (marked with filled circles) and for the entire PTVs (marked with X's). It was found that the PCIs for two targets were significantly lower than for the others (marked with open circle), possibly resulting from an exceptionally short distance between the two targets (1 cm). Stanley *et al*. showed that PCI increased as the tumor volume increased, reaching a saturation value of 0.7 [13]. Our results indicate that the PCI is >0.5 when the tumor volume is >0.1 cm^3^, and the PCI reaches nearly 0.7 when the tumor volume approaches 1.0 cm^3^, suggesting that the 5 mm width MLC may be sufficiently minute for small targets with diameters of <1 cm when multi-arc non-coplanar VMAT SRS is adopted.

**Fig. 7.**
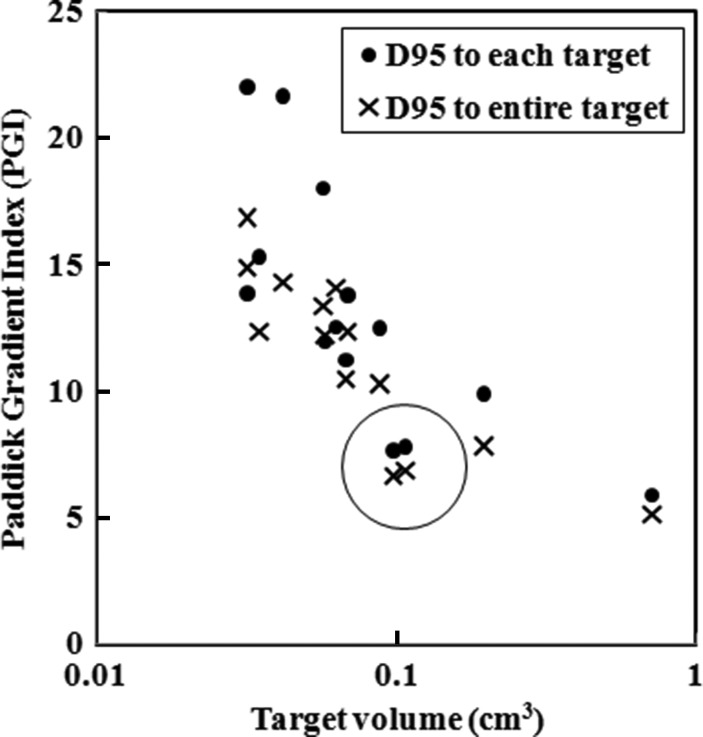
Plots of Paddick's gradient index (PGI) of the 14-mets plan as a function of the target volume, where D_95_ was prescribed for each of the PTVs (marked with filled circles) and to the entire PTVs (marked with X's). Again, PGIs for two targets were significantly lower than others (marked with open circle), possibly resulting from an exceptionally short distance between the two targets (1 cm).

Figure 7 demonstrates plots of the PGI of the 14-mets plan as a function of the target volume, where D_95_ was prescribed for each of the PTVs (marked with filled circles) and for the entire PTVs (marked with X's). Again, PGIs for two targets were significantly lower than for the others (marked with open circle), possibly resulting from an exceptionally short distance between the two targets (1 cm). The dose gradient tends to decrease as the target volume increases [14].

In Table 1, the cause of the larger dose discrepancy in the pinpoint chamber measurement may be due to statistical uncertainty of the Monte Carlo calculation. In other words, an isotropic dose grid of 2 mm results in only a few grids inside the pinpoint chamber volume, whereas the Farmer chamber volume has dose grids >50.

In Table 2, the pass rate of 2%/2 mm at the coronal film position 0 mm is worse than the pass rates on other planes. The plane had one target with a much larger low-dose area. If the dose threshold is changed from 30% to 50% of the maximum measured dose on the plane, the pass rate under the 2%/2 mm criteria is improved from 86.5% to 96.7%.

## CONCLUSION

In conclusion, it was confirmed that dose verification of single-isocenter, non-coplanar multi-arc VMAT radiosurgery for multiple brain metastases is feasible using an Elekta Synergy with Agility and Monaco treatment-planning systems. It was further shown that film dosimetry was accurately performed for a dose of up to nearly 25 Gy.

## CONFLICT OF INTEREST

S.O. has a consulting contract with Elekta KK.
